# Supplementary material for: Learning curve and surgical outcome of robotic assisted colorectal surgery with ERAS program
Source: Sci Rep. 2022 Nov 29;12:20566. doi: 10.1038/s41598-022-24665-w (PMC9709162; doi:10.1038/s41598-022-24665-w)
Supplement: Supplementary file 1 — Supplementary Information 1. [file 41598_2022_24665_MOESM1_ESM.zip › LSC Raw data-Ñ[▒K/086/P_ñw╝╨░O▒Kñσ.pdf]

臺中榮民總醫院 臺中市臺灣大道四段1650號 病理部活體組織切片 臨床醫學實驗及病理診斷中心 檢驗(查)報告

[redacted]

Pathologic diagnosis:
Colon, transverse to descending, laparoscopic left hemocolectomy ---
Focal superficial mucosal necrosis, consistent with ischemic change.
Bialteral cut ends are viable.

Ancillary study for diagnosis:
IHC stain for CMV (-) done for section E.

Gross description:
The specimen consists of a segment of colon, 23 cm in length, 5 an 6 cm in circumference of bilateral cut ends. Some parts of colon show mucosa erosion. No tumor seen. The distal and proximal resection margins are grossly viable. Representative parts for section: A-B) bilateral cut ends C) vascular cut end D) mesentery E-H) mucosa.

Microscopic description:
Sections show colon mucosa with focal superficial mucosa necrosis.
#T-59200_2 #F-39340_2 0 1100 000000

已電子簽章

敬告：本單僅供醫師參考使用，不具任何申請效力！！
請病患妥為保管，若不慎遺失需自負個人資料外洩責任！
